# Analysis of motor dysfunction in Down Syndrome reveals motor neuron degeneration

**DOI:** 10.1371/journal.pgen.1007383

**Published:** 2018-05-10

**Authors:** Sheona Watson-Scales, Bernadett Kalmar, Eva Lana-Elola, Dorota Gibbins, Federica La Russa, Frances Wiseman, Matthew Williamson, Rachele Saccon, Amy Slender, Anna Olerinyova, Radma Mahmood, Emma Nye, Heather Cater, Sara Wells, Y. Eugene Yu, David L. H. Bennett, Linda Greensmith, Elizabeth M. C. Fisher, Victor L. J. Tybulewicz

**Affiliations:** 1 The Francis Crick Institute, London, United Kingdom; 2 UCL Institute of Neurology, London, United Kingdom; 3 Wolfson Centre for Age-Related Diseases, Kings College London, London, United Kingdom; 4 MRC Harwell Institute, Harwell Campus, Oxfordshire, United Kingdom; 5 The Children’s Guild Foundation Down Syndrome Research Program, Genetics Program and Department of Cancer Genetics, Roswell Park Cancer Institute, Buffalo, NY, United States of America; 6 Nuffield Department of Clinical Neurosciences, University of Oxford, Oxford, United Kingdom; 7 Imperial College, London, United Kingdom; IGBMC/ICS, FRANCE

## Abstract

Down Syndrome (DS) is caused by trisomy of chromosome 21 (Hsa21) and results in a spectrum of phenotypes including learning and memory deficits, and motor dysfunction. It has been hypothesized that an additional copy of a few Hsa21 dosage-sensitive genes causes these phenotypes, but this has been challenged by observations that aneuploidy can cause phenotypes by the mass action of large numbers of genes, with undetectable contributions from individual sequences. The motor abnormalities in DS are relatively understudied—the identity of causative dosage-sensitive genes and the mechanism underpinning the phenotypes are unknown. Using a panel of mouse strains with duplications of regions of mouse chromosomes orthologous to Hsa21 we show that increased dosage of small numbers of genes causes locomotor dysfunction and, moreover, that the *Dyrk1a* gene is required in three copies to cause the phenotype. Furthermore, we show for the first time a new DS phenotype: loss of motor neurons both in mouse models and, importantly, in humans with DS, that may contribute to locomotor dysfunction.

## Author summary

Down Syndrome is caused by an extra copy of chromosome 21 and results in many different phenotypes including learning difficulties, Alzheimer’s disease and problems with motor function such as abnormal gait and poor fine motor skills. These different phenotypes are thought to result from an increased copy of one more of the genes on chromosome 21, but it is not known which gene or genes cause the phenotypes. Using a panel of mouse strains with an extra copy of different sets of mouse genes that are equivalent to the human genes on chromosome 21 we were able to show that an extra copy of a small number of genes was sufficient to cause motor abnormalities in the mice, and that one of these genes, *Dyrk1a*, was required in three copies for this phenotype. Furthermore, we found that these mouse models of Down Syndrome showed loss of motor neurons, which could account in part for the motor dysfunction. This result led us to look in spinal cords from humans with DS, where we also found decreased numbers of motor neurons, a phenotype that has never been previously reported.

## Introduction

Down Syndrome (DS), trisomy 21, is characterized by a wide range of phenotypes including cognitive deficits, early-onset Alzheimer’s disease, locomotor dysfunction and congenital heart defects [1, 2]. These diverse phenotypes could be caused by small numbers of dosage-sensitive genes on Hsa21 whose increased copy number leads to increased expression and hence phenotypic effects [3]. Alternatively, phenotypes may result from increased dosage and thus expression of large numbers of sequences on Hsa21. Such a possibility was recently reported for aneuploidy in yeast, where the deleterious effects of an extra chromosome on cell proliferation were shown to be due to the combined action of large numbers of genes rather than small numbers of dosage-sensitive genes, potentially resulting in proteotoxic stress [4, 5]. Finally, it is possible that aneuploidy per se, rather than additional copies of genes may cause phenotypes; and any combination of these phenomena may be important for individual phenotypes.

**Fig 1:**
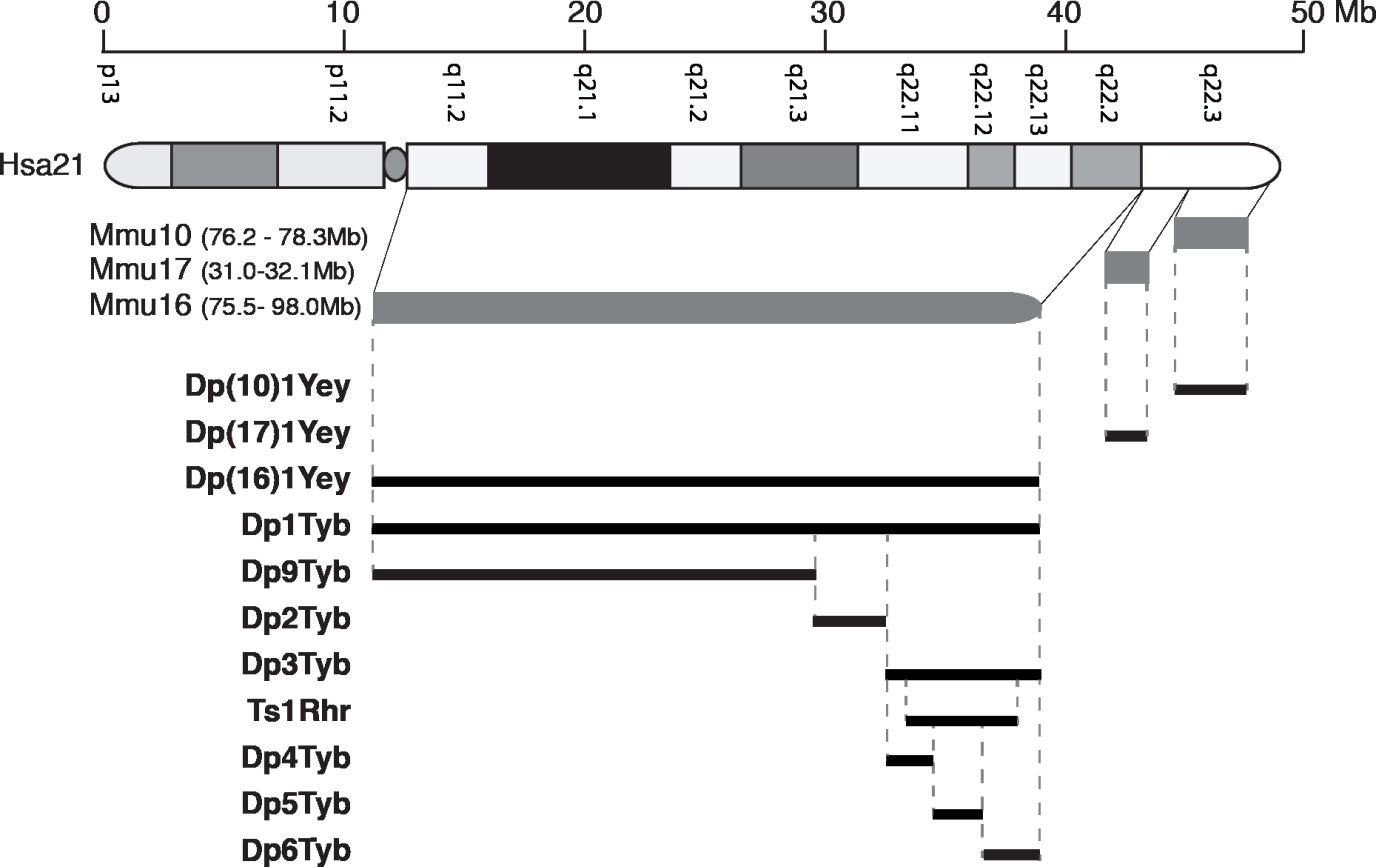
Mouse models of Down syndrome. Diagram at top shows Hsa21 indicating short and long arms separated by the centromere (oval), banding structure and length in Mb. Below this are shown the orthologous regions on Mmu10, Mmu17 and Mmu16 (grey) with genomic coordinates (mouse assembly GRCm38/mm10), and then mouse models used in the current study indicating the regions duplicated in tandem in each model (black).

To understand the genetic basis of DS, we and others have used mouse genetics to model the syndrome by generating a series of strains containing either Hsa21 or duplications of mouse chromosome regions orthologous to Hsa21 located on mouse chromosome 16 (Mmu16), Mmu17 and Mmu10 (**Fig 1**) [1, 6–13]. Some of these strains are also aneuploid–the Tc1 mouse strain carries a freely segregating Hsa21 and has increased dosage of genes on Hsa21 [9]. While Tc1 mice have a number of phenotypes that model aspects of DS, the mice are mosaic for Hsa21 and this chromosome is rearranged such that only ~200 Hsa21 protein-coding genes are present (~75% of all Hsa21 genes) [14]. The Ts65Dn strain is also aneuploid, containing an extra chromosome carrying a portion of Mmu16 orthologous to Hsa21 and has been used extensively for the study of DS phenotypes [7]. However, this additional chromosome also includes ~10 Mb of Mmu17 containing 60 mouse genes that are not orthologous to Hsa21, thus limiting the utility of this model [15]. Chromosome engineering has allowed the generation of strains with carefully designed gene dosage increases. In particular, three strains known as Dp(16)1Yey, Dp(17)1Yey and Dp(10)1Yey, have been generated carrying tandem duplications of the entire Hsa21-orthologous regions of Mmu16, Mmu17 and Mmu10 respectively (**Fig 1**) [10, 11]. The intercross of these three mutations (triple trisomic mouse) gives the most complete mouse model of DS to date because it carries an extra copy of every mouse gene orthologous to Hsa21. We have also generated the Dp1Tyb strain with a tandem duplication of the entire region of Mmu16 orthologous to Hsa21, the largest of the three syntenic regions [16]. Furthermore, to allow an unbiased mapping of dosage-sensitive genes that may cause DS phenotypes we constructed another 6 strains (Dp2Tyb, Dp3Tyb, Dp4Tyb, Dp5Tyb, Dp6Tyb and Dp9Tyb) that have duplications of shorter regions of Mmu16 nested within the region duplicated in Dp1Tyb (**Fig 1**) [16]. Thus, between them, these strains can be used to establish if phenotypes are caused by small or large numbers of genes, or they if they require aneuploidy.

People with DS have deficits in motor function, showing alterations in balance, postural control and fine motor skills [17–22]. This understudied DS phenotype has been suggested to arise in part because of defects in cerebellar anatomy [23, 24]. The Ts65Dn mouse model has reduced cerebellar size and decreased numbers of granule cells, and the same defects were found in cerebella of humans with DS [24]. The defect in this model is likely caused by decreased Sonic Hedgehog-induced proliferation of granule cell precursors [25]. However, we do not know the identity of any dosage-sensitive genes whose increased copy number is required for motor defects, or indeed if these defects are caused by small numbers of dosage-sensitive genes, or by the mass action of increased dosage of large numbers of genes on Hsa21, or by aneuploidy.

In this study, we aimed to identify dosage-sensitive genes that cause motor defects and to examine possible pathological changes that might underpin them. In particular, we investigated changes in cerebellar anatomy as well as sensory and motor neuron function. Using our genetic mapping panel of mouse strains, we show that motor dysfunction can be caused by increased dosage of a region with a small number of genes, and within these we demonstrate that the *Dyrk1a* gene is required in three copies to cause the phenotype. Furthermore, we show that, surprisingly, there is no alteration in cerebellar anatomy in mice that have increased dosage only of genes orthologous to Hsa21. However, we identified an entirely novel form of neurodegeneration in DS, the progressive loss of motor neurons, a phenotype that, importantly, is recapitulated in human samples with DS and may contribute to the locomotor dysfunction. Our results support the hypothesis that some DS phenotypes are caused by increased copy number of small numbers of dosage-sensitive genes, and broaden the neurodegenerative phenotypes in DS.

## Results

### Locomotor dysfunction is caused by increased dosage of a genes within a 3.3 Mb region

**Fig 2:**
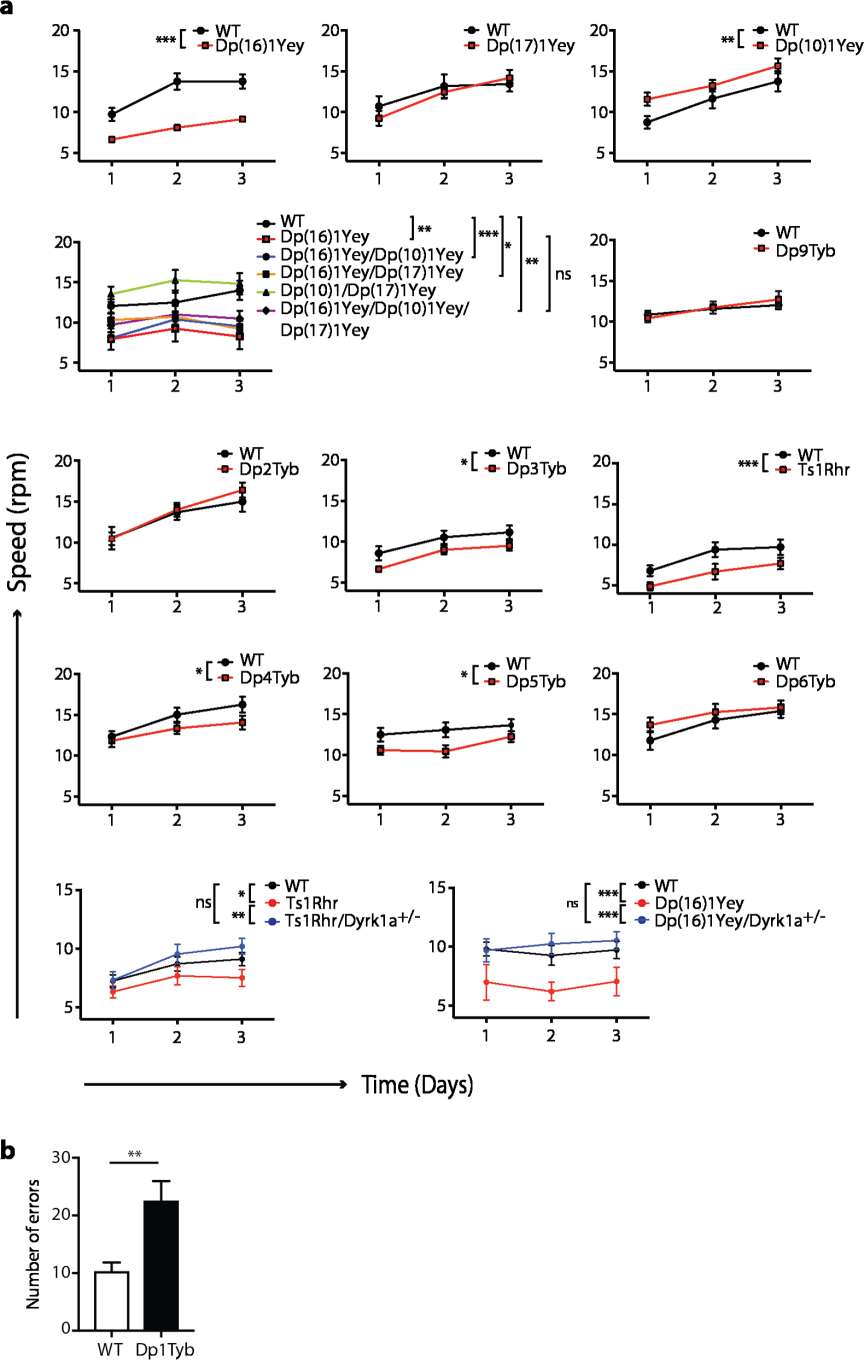
Locomotor dysfunction is caused by increased dosage of two short regions of Mmu16 including *Dyrk1a*. (**a**) Graphs show performance of 12-week old mice of the indicated genotypes on an accelerating Rotarod, showing the mean±SEM speed (rpm) at which they fell off, on three successive days of testing (n = 12 wild-type[WT], 14 Dp(16)1Yey; 11 WT, 12 Dp(17)1Yey; 11 WT, 17 Dp(10)1Yey; 6 WT, 5 Dp(16)1Yey, 6 Dp(16)1Yey/Dp(17)1Yey, 6 Dp(16)1Yey/Dp(10)1Yey, 10 Dp(17)1Yey/Dp(10)1Yey, 6 Dp(16)1Yey/Dp(17)1Yey/ Dp(10)1Yey; 13 WT, 15 Dp9Tyb; 9 WT, 12 Dp2Tyb; 9 WT, 18 Dp3Tyb; 15 WT, 15 Ts1Rhr; 15 WT, 15 Dp4Tyb; 15 WT, 20 Dp5Tyb; 15 WT, 15 Dp6Tyb; 36 WT, 18 Ts1Rhr, 21 Ts1Rhr/*Dyrk1a*^+/-^; 10 WT, 5 Dp(16)1Yey, 10 Dp(16)1Yey/*Dyrk1a*^+/-^). Data analyzed with 2-way ANOVA, *p < 0.05, **p < 0.01, ***p < 0.001; ns, not significant. (**b**) Mean±SEM number of foot errors made by 11-week old mice traversing a horizontal ladder in a Locotronic apparatus (n = 13 WT, 12 Dp1Tyb). Data analyzed with unpaired t-test, **p < 0.01.

Previously, we showed that Tc1 mice have defects in locomotor function using both a static rod and a rotating rod (Rotarod) test [26]. To identify whether locomotor defects can be modeled by increased dosage of mouse genes orthologous to Hsa21, we examined locomotor function in Dp(16)1Yey, Dp(17)1Yey and Dp(10)1Yey mice that between them carry duplications of all three regions of mouse chromosomes orthologous to Hsa21 (**Fig 1**). We chose to use a Rotarod paradigm in which mice are placed onto an accelerating rod, recording the speed of the rod at which the mouse falls. Each mouse was tested 3 times during one day, and then a further 3 times on the second and third day of testing–a protocol in which control mice usually show improved performance over the 3 days, demonstrating motor learning. We found Dp(16)1Yey mice performed significantly less well than wild-type littermates, whereas Dp(17)1Yey mice showed no defects, and Dp(10)1Yey mice had improved performance, demonstrating that duplication of the orthologous region on Mmu16 was sufficient to cause locomotor defects (**Fig 2A**). To evaluate whether the orthologous regions on Mmu17 and Mmu10 may contribute to the phenotype when combined with the duplication on Mmu16, we intercrossed the three mutant strains and analyzed the progeny. We found that the Dp(17)1Yey and Dp(10)1Yey mutations alone or together did not exacerbate the phenotype of the Dp(16)1Yey mice, with the triple trisomic mice performing no worse than Dp(16)1Yey (**Fig 2A**). Thus, the region of Hsa21 orthology on Mmu16 is both required and sufficient to cause locomotor defects.

It is possible that the mice performed poorly in the Rotarod test because of reduced motivation rather than locomotor dysfunction. To address this, we tested Dp1Tyb mice (which bear a duplication of the same Mmu16 genes as Dp(16)1Yey mice) using the Locotronic apparatus (Intellibio). In this assay, mice traverse a horizontal ladder with evenly spaced rungs, and the number of errors in foot placement (missed rungs) was recorded. Mice were tested 2–3 times, and trials where mice took > 60 s to traverse the ladder were excluded from the analysis in order to eliminate trials where the mice were insufficiently motivated. Dp1Tyb mice showed a significant increase in errors, supporting the conclusion that duplication of the Hsa21-orthologous region of Mmu16 results in locomotor defects (**Fig 2B**).

To narrow down the location of potential dosage-sensitive genes causing this defect, we examined Dp2Tyb, Dp3Tyb and Dp9Tyb mice which contain duplications that between them cover the entire region duplicated in Dp(16)1Yey (**Fig 1**). We found that only Dp3Tyb mice had a significant defect in the Rotarod assay, though we noted that the extent of the defect was smaller than that seen in Dp(16)1Yey (**Fig 2A**). A similar defect was also seen in Ts1Rhr mice that contain a duplication of 33 genes that is entirely contained within the Dp3Tyb region, but is smaller by 8 genes [27]. Lastly, we examined Dp4Tyb, Dp5Tyb and Dp6Tyb mice that break down the region duplicated in Dp3Tyb into three smaller regions. We found both Dp4Tyb and Dp5Tyb showed defects in the Rotarod assay. Thus, the regions duplicated in Dp4Tyb and Dp5Tyb, spanning a total of 3.3Mb and containing 15 and 12 genes respectively are each sufficient to cause some locomotor dysfunction, although genes in other areas also contribute to the full phenotype.

### *Dyrk1a* is required in three copies for locomotor dysfunction

DYRK1A is protein kinase encoded on Hsa21, whose overexpression has been implicated in neuronal phenotypes in DS, including brain development and synaptic plasticity [28]. Transgenic mice overexpressing DYRK1A have motor defects [29–32], and since *Dyrk1a* is located within the region duplicated in Dp(16)1Yey, Dp3Tyb, Ts1Rhr and Dp5Tyb mice, we tested whether three copies of *Dyrk1a* are required for the locomotor defects. We crossed Dp(16)1Yey and Ts1Rhr mice to mice heterozygous for a null allele of *Dyrk1a* (*Dyrk1a*^+/-^). The phenotype was rescued in both the Dp(16)1Yey/*Dyrk1a*^+/-^ and Ts1Rhr/*Dyrk1a*^+/-^ progeny (2 copies of *Dyrk1a*), which showed no defect in the Rotarod assay, thus demonstrating that three copies of the *Dyrk1a* gene are required for the locomotor deficit (**Fig 2A**).

**Fig 3:**
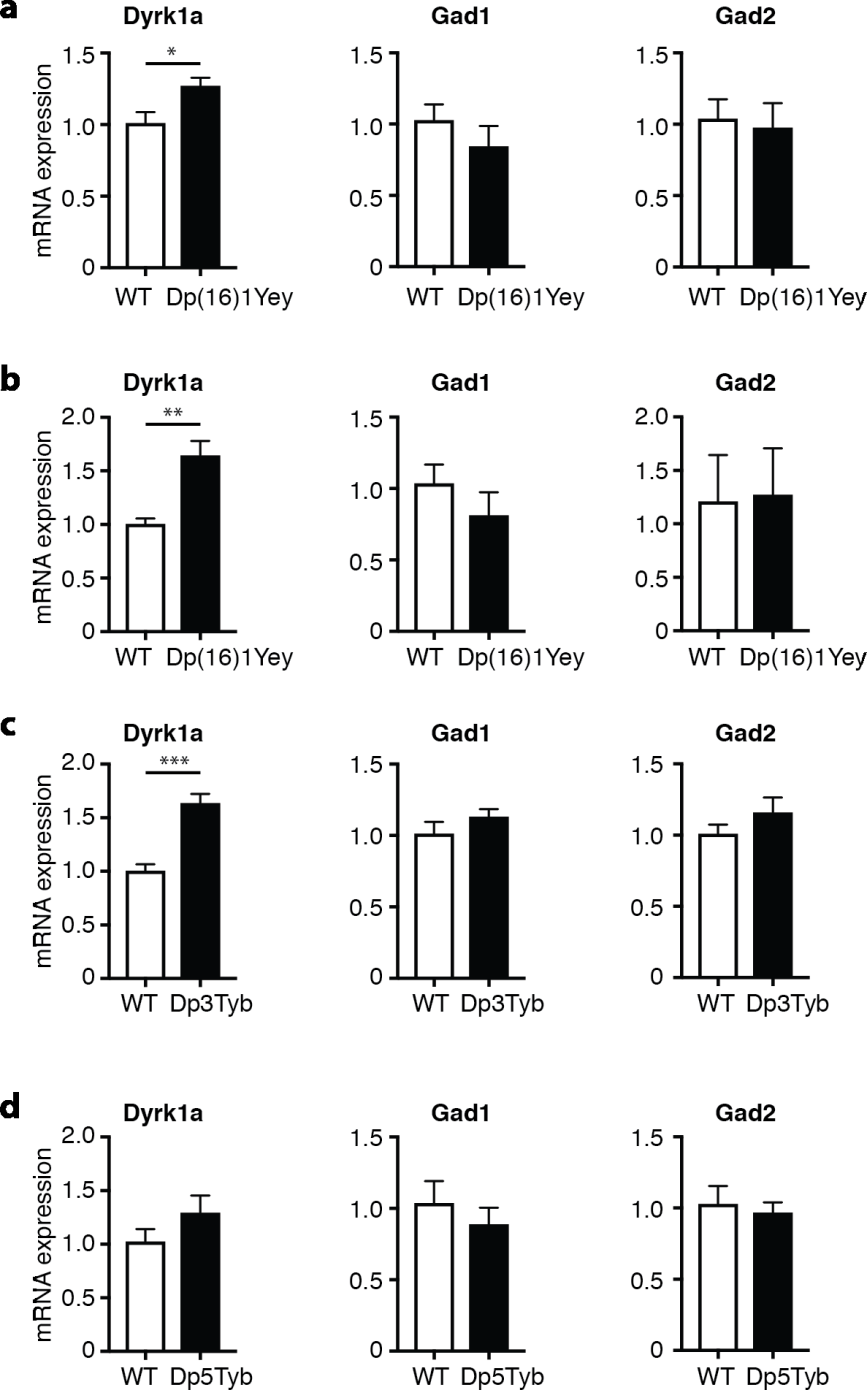
Increased *Dyrk1a* expression in Dp(16)1Yey and Dp3Tyb mice. (**a-d**) Mean±SEM mRNA levels of *Dyrk1a*, *Gad1* and *Gad2* in the cerebellum of Dp(16)1Yey mice at 10 weeks (**a**) and 6 days (**b**) of age and in 10 week old Dp3Tyb (**c**) and Dp5Tyb (**d**) mice. Expression of the test gene was normalized to *Gapdh* and then to expression in WT control mice (n = 5 of each genotype). Data analyzed with unpaired t-test, *p < 0.05, **p < 0.01, ***p < 0.001.

If an increased gene dosage of *Dyrk1a* is required for the locomotor defects, there should be increased *Dyrk1a* mRNA expression in mice with a duplication that includes this gene. We found significantly increased *Dyrk1a* mRNA in the cerebellum of Dp(16)1Yey mice at both 6 days and 10 weeks of age and in 10 week old Dp3Tyb mice (**Fig 3A–3C**). We also saw a trend for increased *Dyrk1a* expression in Dp5Tyb mice (**Fig 3D**). Interestingly, the upregulation of *Dyrk1a* was larger in young Dp(16)1Yey mice at 6 days of age (1.64-fold) compared to adult 10 week old mice (1.25-fold). This is similar to a previous report on cerebellar DYRK1A levels in Ts1Cje mice that have an additional copy of 87 Mmu16 genes including *Dyrk1a* [33]. This study shows that the increase in cerebellar DYRK1A was larger in young compared to old Ts1Cje mice.

It has been previously described that increased *Dyrk1a* expression in transgenic mBACtgDyrk1a mice leads to increased levels of *Gad1* and *Gad2* in several brain regions including the cerebellum [34]. GAD1 and GAD2 are isoforms of glutamate decarboxylase, an enzyme that synthesizes the gamma-aminobutyric acid (GABA) neurotransmitter, and are expressed in inhibitory interneurons. The increased levels of GAD1 and GAD2 in mice overexpressing *Dyrk1a* have been postulated to indicate elevated numbers of inhibitory neurons, a phenotype that could contribute directly to behavioral and cognitive changes [34]. Analysis of cerebellar mRNA in Dp(16)1Yey mice at 6 days and 10 weeks of age and in 10-week old Dp3Tyb and Dp5Tyb mice showed no significant change in the expression of either *Gad1* or *Gad2* (**Fig 3A–3D**). This result shows that the upregulation of *Dyrk1a* in these strains (1.25- to 1.64-fold) is insufficient to perturb expression of *Gad1* or *Gad2*, and suggests that numbers of interneurons may not be altered in the cerebellum of these mice.

### No alteration in cerebellar anatomy in Dp(16)1Yey mice

**Fig 4:**
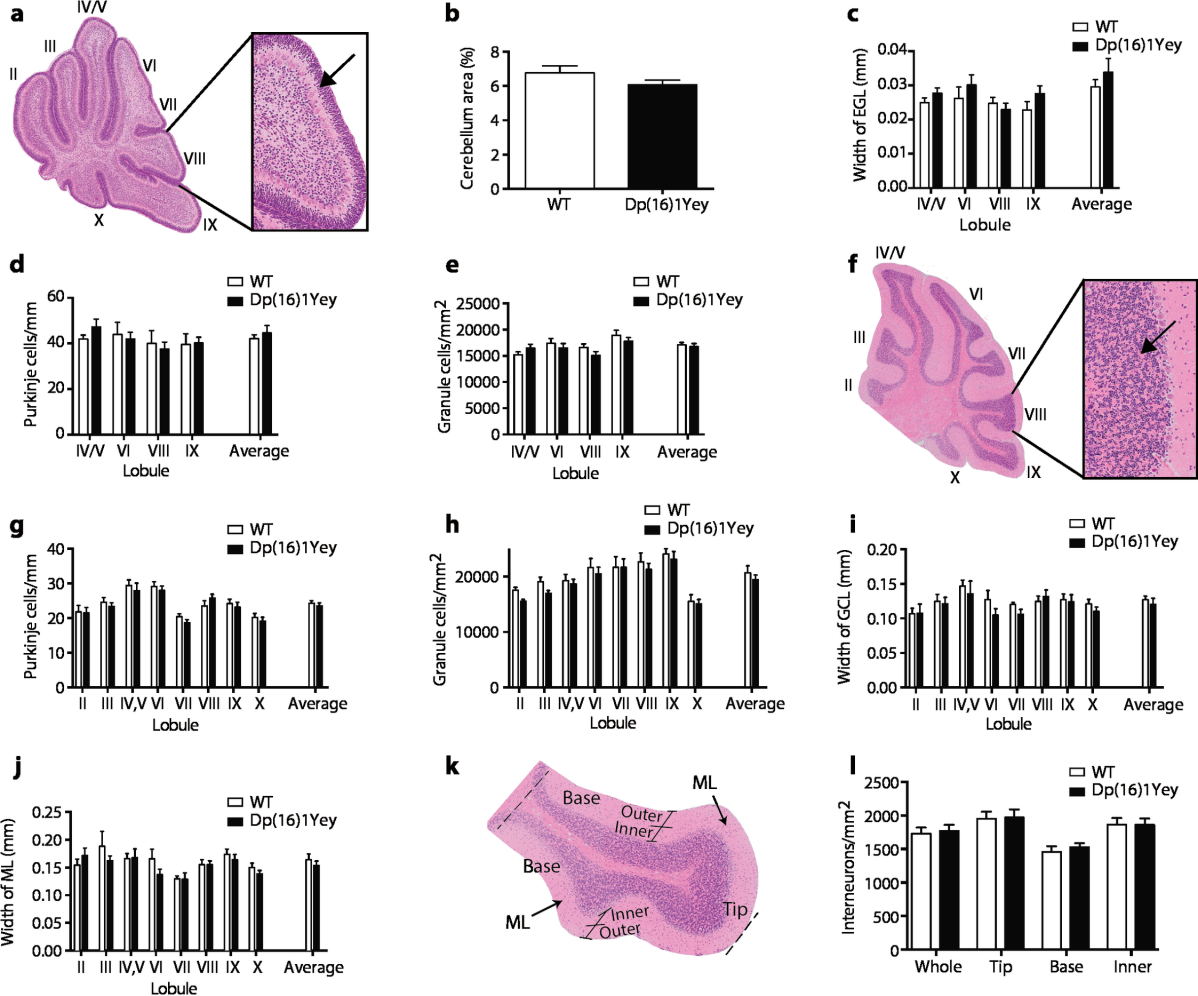
Normal cerebellar anatomy in Dp(16)1Yey mice. (**a**-**e**) Analysis of cerebellar anatomy in Dp(16)1Yey mice at P6. (**a**) Cross section through cerebellum stained with H&E with expanded view showing small purple granule cells in the external granule layer (EGL) (arrow), Roman numerals indicate lobules, (**b**) area of cerebellum as % of whole brain, (**c**) width of the EGL, (**d**) linear density of Purkinje cells, (**e**) density of granule cells in the EGL. (**f**-**l**) Analysis of cerebellar anatomy in Dp(16)1Yey mice at 6 months of age. (**f**) Cross section through cerebellum stained with H&E with expanded view showing small purple granule cells in the granule cell layer (GCL) (arrow), Roman numerals indicate lobules, (**g**) linear density of Purkinje cells, (**h**) density of granule cells in the GCL, (**i**) width of the GCL, (**j**) width of the molecular layer (ML), (**k**) cross section of lobule IX showing the ML subdivided into base, tip and inner and outer regions, (**l**) density of interneurons in the whole ML of lobule IX or in the base, tip or inner regions subdivided as in **k**. In **b**-**d** and **g**-**j** values are shown for individual lobules as indicated and also averaged over all lobules analyzed (n = 9 WT, 13Ts(16)1Yey P6 whole brain area; n = 7 WT, 12 Dp(16)1Yey at P6 for all other measurements; n = 9 WT and Dp(16)1Yey at 6 months). All graphs show mean±SEM.

Since defects in cerebellar anatomy have been described in both humans with DS and in the aneuploid Ts65Dn and Tc1 mouse models [9, 24], and these have been proposed to contribute to motor defects in DS [23], we investigated the anatomy of the cerebellum in Dp(16)1Yey mice, which have motor defects but are not aneuploid. We analyzed cerebella at two ages, 6 days after birth (P6) and in adult mice at 6 months of age, since previous studies had documented changes at these ages in Ts65Dn mice [24, 25]. At P6 we found no significant changes in cerebellar area, width of the external granule cell layer, or in the density of Purkinje cells or granule cells in Dp(16)1Yey mice, either in individual lobules or averaged over the whole cerebellum (**Fig 4A–4E**). Similarly, in adult mice there was no change in the width of the granule cell and molecular layers or in the density of Purkinje cells, granule cells or interneurons, again over individual lobules or averaged over the whole structure (**Fig 4F–4L**). Thus, in contrast to Ts65Dn mice, Dp(16)1Yey mice have no obvious defect in cerebellar anatomy, and this cannot contribute to their locomotor dysfunction. These results do not rule out that there may be functional abnormalities in the cerebellum despite the absence of anatomical changes.

### No broad defect in sensory function in Tc1 and Dp1Tyb mice

**Fig 5:**
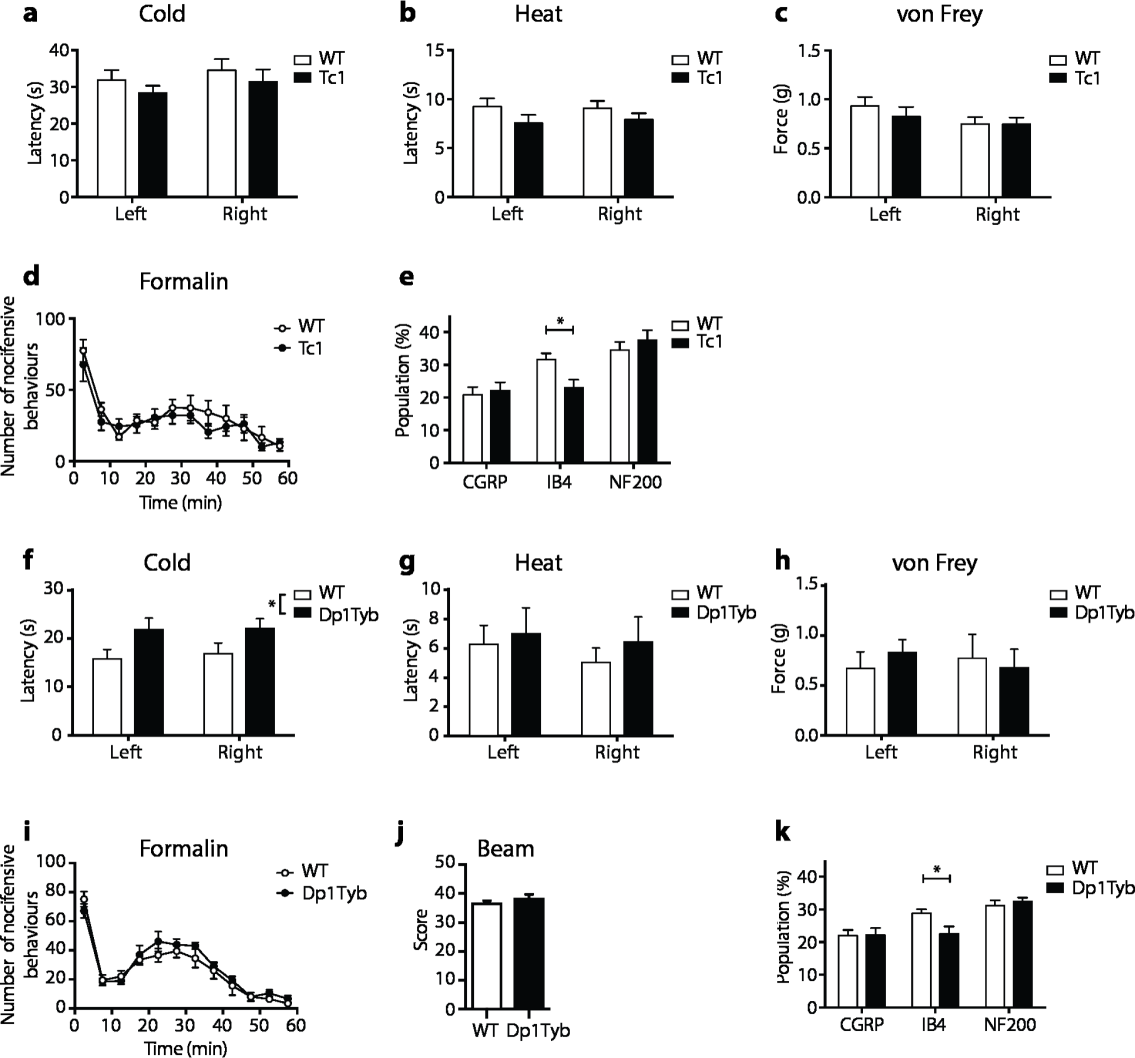
No broad defect in sensory behavior in Tc1 or Dp1Tyb mice. Analysis of Tc1 (**a**-**e**) and Dp1Tyb (**f-k**) mice at 14 weeks (**a-d**, **f-j**), or 11 weeks (**e,k**). (**a,f**) Withdrawal latency of right and left hind paws in response to cold plate. (**b,g**) Withdrawal latency of right and left hind paws in response to radiant heat (Hargreaves test). (**c,h**) Force required for 50% withdrawal of right and left hind paws in response to punctate mechanical stimulation (Von Frey test). (**d,i**) Number of nocifensive behaviors as a function of time following formalin injection into hind paw. (**e**,**k**) Quantification of sensory neuron subpopulations in dorsal root ganglia taken from L3-L5 region of (**e**) Tc1 mice and (**k**) Dp1Tyb mice showing the proportion of DRG cell profiles positive for the indicated markers. (**j**) Score representing ability of WT or Dp1Tyb mice to walk up a tapered inclined beam; a higher score indicates better performance. All graphs show mean±SEM (**a-d**, n = 10; **e**, n = 5; **f-j**, n = 7; **k**, n = 5). Data analyzed with unpaired t-test, *p < 0.05.

Given the changes in motor function we also screened for sensory deficits across a range of modalities, since these can result in locomotor defects [35, 36], and have not been examined in DS. Using assays measuring nocifensive responses to cold, heat, mechanical stimulation and formalin injection, we compared Tc1 mice with controls and found no differences between the groups (**Fig 5A–5D**). We also analyzed the dorsal root ganglia (DRG) by histology. Sensory neuron cell bodies located in the DRG can be classified based on their neurochemical characteristics. Large and medium diameter sensory neurons with myelinated axons were identified by expression of NF200. Peptidergic or non-peptidergic small diameter sensory neurons with unmyelinated axons (C-fibers) were identified by expression of calcitonin gene related peptide (CGRP) or by binding the isolectin *Griffonia simplicifolia* I-B4 (IB4) respectively [37]. We found no change in the fraction of neurons expressing NF200 or CGRP, but a significant decrease in the fraction of neurons binding IB4 in Tc1 mice (**Fig 5E**). We extended these studies to Dp1Tyb mice that, similar to Dp(16)1Yey mice, contain a duplication of the entire Hsa21 orthologous region on Mmu16 (**Fig 1**). Once again we found no changes in nocifensive responses to heat, mechanical stimulation and formalin injection, but the response to cold was partially impaired (**Fig 5F–5I**). Importantly, Dp1Tyb mice showed no defect in a beam walk test, a measure of proprioception (**Fig 5J**), but, interestingly, we again found a decrease in IB4-binding neurons in the DRGs of Dp1Tyb mice, similar to that seen in Tc1 mice (**Fig 5K**). These IB4+ neurons respond to high threshold noxious mechanical stimuli and thus their reduction is unlikely to contribute to the locomotor defects [38]. In summary, we found little evidence of a broad sensory defect in mouse models of DS that could account for the motor defects, but discovered a specific reduction in one class of nociceptive sensory neurons, IB4+ afferents, in Tc1 and Dp1Tyb mice, a phenotype that would merit further investigation in both mice and humans.

### Degenerative loss of motor neurons and motor function in Tc1 mice

**Fig 6:**
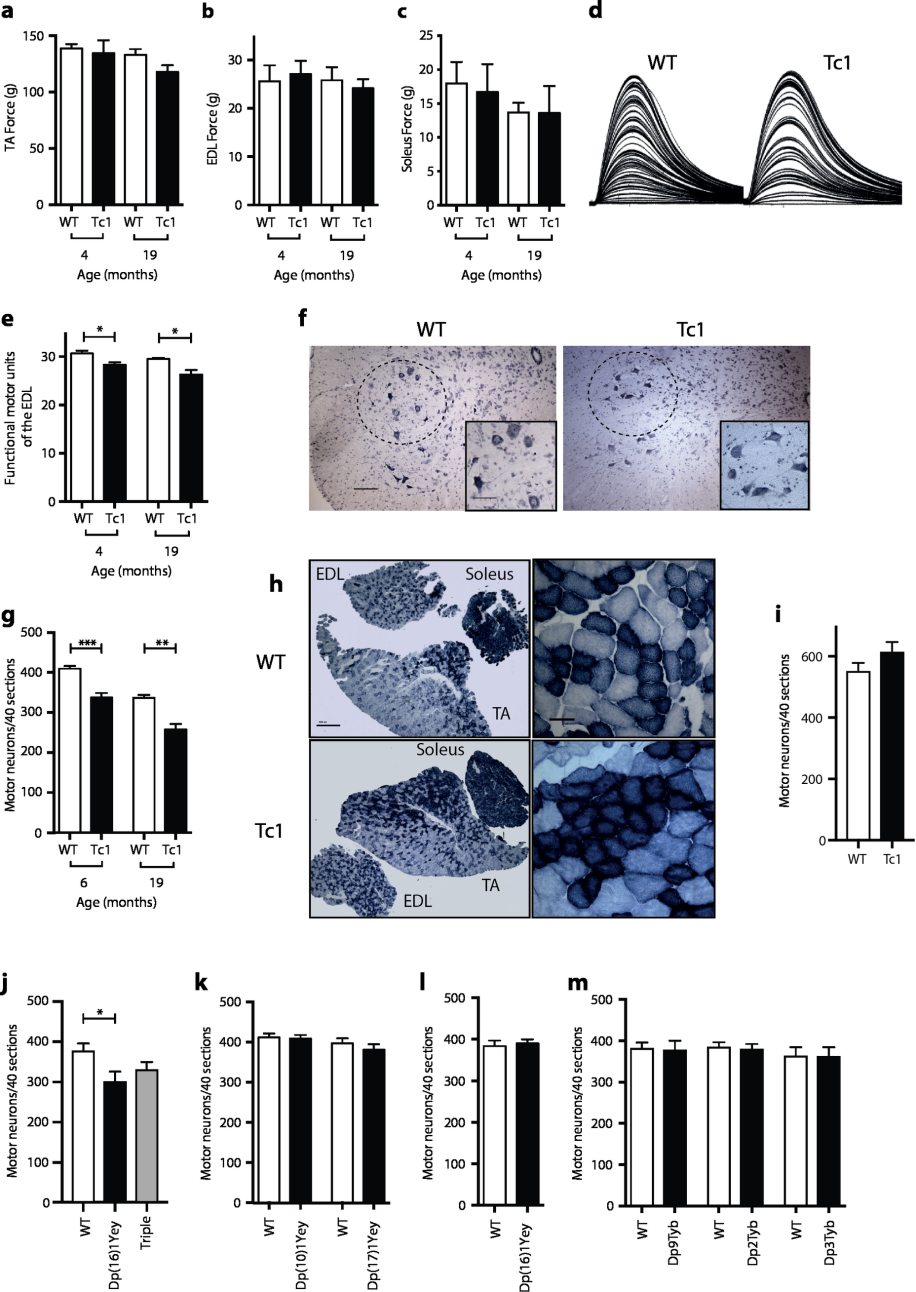
Decreased numbers of motor neurons in Tc1 and Dp(16)1Yey mice. (**a**-**e**) Analysis of Tc1 mice and WT littermate controls at the indicated ages (n = 4 WT, 5 Tc1 at 4 months; 4 WT, 5 Tc1 at 19 months). Maximum force production by (**a**) TA, (**b**) EDL, and (**c**) Soleus muscles in response to tetanic stimulation. (**d**) Typical motor unit recording traces in WT and Tc1 mice, showing stepwise increments in single twitch force production in response to increased stimulating intensity. The number of increments recorded is taken as the number of motor units. (**e**) Number of motor units in EDL muscles of indicated mice. (**f**) Nissl-stained lumbar spinal cord sections of WT and Tc1 mice at 19 months of age, with the sciatic motor pool indicated by a dashed line. Inset shows motor neurons in the sciatic pool. Scale bars, 100 μm, inset 50 μm. (**g**) Number of spinal cord motor neurons in WT and Tc1 mice at 6 months (n = 8 WT, 5 Tc1) and 19 months of age (n = 4 WT, 5 Tc1). (**h**) Sections of the EDL, TA and soleus muscles of WT and Tc1 mice at 19 months of age, stained for succinate dehydrogenase (SDH) at low (left) and high (right) magnification. Muscle fibers with dark blue staining have higher SDH activity and thus rely on oxidative phosphorylation and are more likely to be slow fibers. Clustering of darker fibers in Tc1 mice indicates denervation and subsequent reinnervation by slow motor neurons and a shift towards slower muscle fibers. Scale bars: low magnification, 500 μm; high magnification, 50 μm. (**i**) Number of motor neurons in WT and Tc1 mice at 22d of age (n = 4 WT, 6 Tc1). (**j, k**) Number of motor neurons in mice of the indicated genotypes and matched controls at 6 months of age (n = 6 WT, 6 Dp(16)1Yey, 8 Dp(16)1Yey/Dp(10)1Yey/Dp(17)1Yey triple trisomic; 7 WT, 7 Dp(10)1Yey; 6 WT, 6 Dp(17)1Yey). (**l**) Number of motor neurons in Dp(16)1Yey mice and controls at P6 (n = 5 WT, 5 Dp(16)1Yey). (**m**) Number of motor neurons in mice of the indicated genotypes and matched controls at 6 months of age (n = 6 WT, 7 Dp9Tyb; 7 WT, 7 Dp2Tyb; 8 WT, 10 Dp3Tyb). All graphs show mean±SEM. Data analyzed with unpaired t-test, *p < 0.05, **p < 0.001, ***p < 0.0001.

We undertook a series of studies to investigate if defects in muscle function or its innervation contribute to the locomotor defects. We previously showed that Tc1 mice have no defect in muscle strength as measured by a grip test [26]. To extend these studies we measured the maximum force produced by the tibialis anterior (TA), extensor digitorum longus (EDL) and soleus muscles of the mouse hindlimb in response to a tetanic stimulation of the sciatic nerve in live anaesthetized mice. We found no change in muscle force in Tc1 mice at either 4 or 19 months of age (**Fig 6A–6C**). However, analysis of the number of physiological motor units innervating the EDL muscle showed a significant 8% decrease in Tc1 mice at 4 months of age, rising to a 11% decrease at 19 months of age (**Fig 6D and 6E**). In agreement with this, analysis of motor neuron numbers in the sciatic motor pool showed an 18% and a 23% decrease in Tc1 mice at 6 and 19 months of age respectively (**Fig 6F and 6G** and **S1 Table**). Histology of the TA muscles confirmed this observation, showing changes characteristic of denervation and subsequent re-innervation, resulting in characteristic fiber type grouping of oxidative fiber types (**Fig 6H**). Interestingly, there was no decrease in motor neuron numbers in young Tc1 mice aged 22 days, indicating that there is no developmental deficit in the generation of these cells (**Fig 6I**). Thus, Tc1 mice show a progressive loss of motor neurons and motor unit function, which could contribute to the locomotor dysfunction.

### Loss of motor neurons is caused by increased dosage of Hsa21 orthologous genes on Mmu16

To investigate the genetic basis of the motor neuron loss, we counted motor neurons in models with duplications of mouse regions orthologous to Hsa21. Analysis of Dp(16)1Yey, Dp(17)1Yey and Dp(10)1Yey mice at 6 months of age showed that only Dp(16)1Yey mice had decreased numbers of motor neurons (20% reduction), similar to that seen in Tc1 mice (**Fig 6J and 6K** and **S1 Table**). Furthermore, mice with duplications of all three regions (triple trisomic) showed a loss of motor neurons that was no greater than that seen in the single mutant Dp(16)1Yey mice (**Fig 6J**). Thus, duplication of the orthologous region of Mmu16 is both necessary and sufficient to cause a reduction of motor neurons, and the Hsa21 orthologous regions on Mmu10 and Mmu17 do not contribute to the phenotype. In contrast we found no change in motor neuron numbers in Dp(16)1Yey mice at P6 (**Fig 6L**), once again showing that the loss of motor neurons is progressive neurodegeneration and not a developmental deficit.

To map the location of potential dosage-sensitive genes that cause this neurodegeneration, we analyzed motor neuron numbers in Dp2Tyb, Dp3Tyb, and Dp9Tyb mice at 6 months of age (**Fig 1**). We saw no change in motor neuron numbers in any of these three strains (**Fig 6M** and **S1 Table**). Thus, the motor neuron loss is caused by an additional copy of at least 2 genes and these are located in 2 or more of the Mmu16 regions duplicated in Dp2Tyb, Dp3Tyb or Dp9Tyb.

### Humans with DS show loss of motor neurons

**Fig 7:**
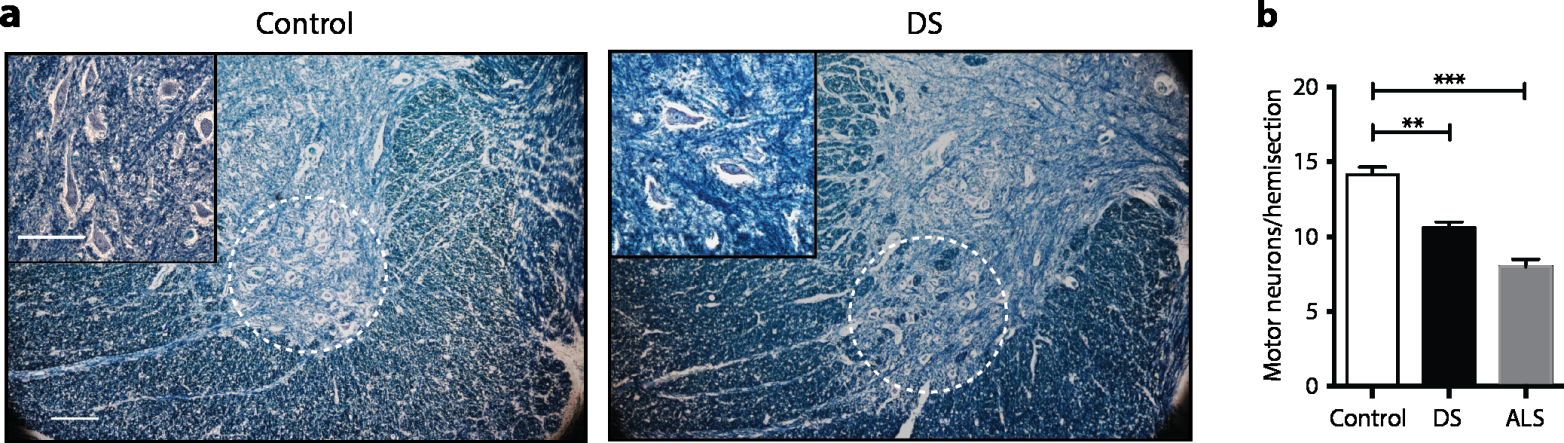
Decreased numbers of motor neurons in humans with DS. (**a**) Examples of human cervical spinal cord sections stained with Luxol Fast Blue. Dashed white circle indicates area containing motor neurons. Inset shows a higher magnification image of the ventral area containing motor neurons. Scale bar, 100 μm. (**b**) Number of motor neurons per hemisection of cervical spinal cord of adults with DS, ALS and controls (n = 4 control, 3 DS, 3 ALS). All graphs show mean±SEM. Data analyzed with unpaired t-test, **p < 0.01, ***p < 0.001.

The loss of motor neurons observed in Tc1 and Dp(16)1Yey mice led us to investigate spinal cord motor neuron numbers in humans with DS, since these have not been previously reported. Strikingly, we found decreased numbers of motor neurons in humans with DS compared to non-DS controls (**Fig 7A and 7B** and **S2 Table**). We compared our results to motor neuron counts in spinal cord sections of people with the motor neuron disease amyotrophic lateral sclerosis (ALS) and found the decrease in DS was less than in ALS. Thus, observation of reduced numbers of motor neurons in a mouse model of DS has led us to discover a novel phenotype in humans with DS.

## Discussion

Using a combination of mouse strains that contain Hsa21 genes or additional copies of their mouse orthologues that are also aneuploid (Tc1) or not (Dp strains) we were able to show that locomotor defects resulted from an additional copy of small numbers of genes, and that aneuploidy was not required for this phenotype. This supports the hypothesis that at least some DS phenotypes are due to increased dosage of small numbers of dosage-sensitive genes, rather than mass action of large numbers of additional genes or aneuploidy. This has important implications for driving forward future investigations into mitigating the effects of a few or single dosage-sensitive genes in DS. Nonetheless we noted that the locomotor defects became weaker as we reduced the size of the duplications, implying that in addition to the small regions containing dosage-sensitive genes that are sufficient on their own to cause phenotypes, there are additional genes outside these regions that also contribute.

The presence of defects in two different locomotor assays (Rotarod and Locotronic) supports our conclusion that the mutant mice performed poorly because they have locomotor defects rather than lacking motivation. In particular, in the Locotronic test we eliminated trials in which the mice took >60 s to traverse the ladder, thereby excluding trials where the mice were insufficiently motivated. However, it is possible that the poorer performance in these tests was due to other causes. For example Dp(16)1Yey have been shown to have disrupted sleep, which could impair performance [39].

**Fig 8:**
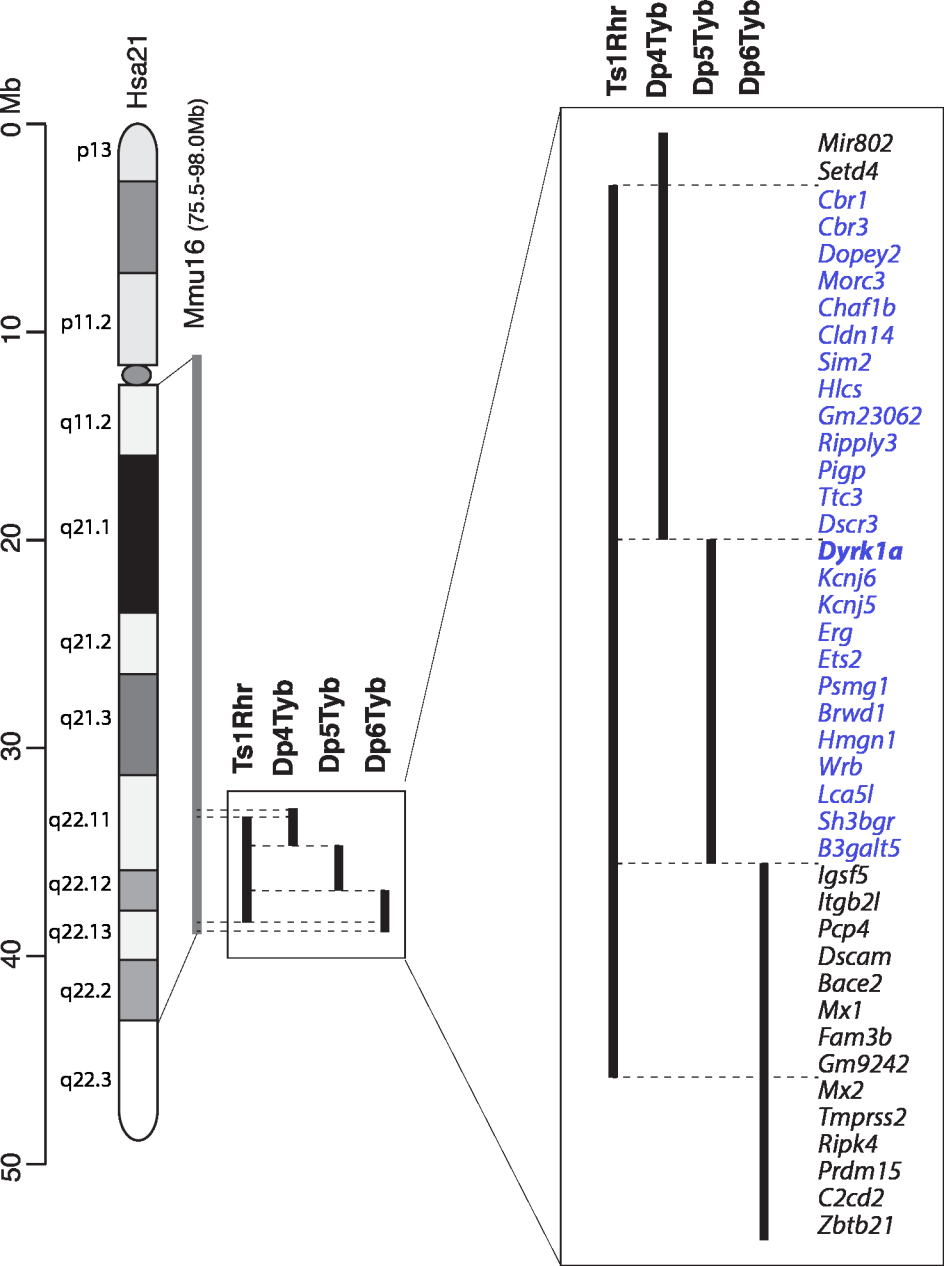
Dosage-sensitive region causing locomotor dysfunction. Diagram on left shows Hsa21 indicating short and long arms separated by the centromere (oval), banding structure and length in Mb. The orthologous region of Mmu16 is indicated in grey and the regions of Mmu16 duplicated in Ts1Rhr, Dp4Tyb, Dp5Tyb and Dp6Tyb mouse models are indicated in black. On the right these duplicated regions are expanded and all known protein coding genes in these intervals and two microRNA genes (*Mir802* and *Gm23062*) are listed (mouse genome assembly GRCm38.p4). The locomotor defect assayed by Rotarod maps to a minimal interval resulting from the overlap of Ts1Rhr, Dp4Tyb and Dp5Tyb with genes in the interval indicated in blue. The *Dyrk1a* gene (bold) is required in 3 copies for the locomotor defect. Genes outside this region are listed in black.

The locomotor defect was evident in both Dp4Tyb and Dp5Tyb mice, showing that increased dosage of two separate regions is sufficient to cause this phenotype, and implies that at least two different dosage-sensitive genes contribute to this defect. It is likely that the more severe phenotype in Dp(16)1Yey mice is caused by additive effects of the Dp4Tyb and Dp5Tyb regions, together with genes outside these regions. Furthermore, since Ts1Rhr mice also have defects, the dosage-sensitive genes are most likely to be within the 25 genes that are duplicated in both Ts1Rhr and Dp4Tyb and Dp5Tyb (**Fig 8**). One of these is *Dyrk1a* and we have shown that three copies of this gene are required for the locomotor defect in Dp(16)1Yey mice, in agreement with previous studies showing that overexpression of DYRK1A leads to motor defects [29–32]. However, our results also show that the situation is complex, since Dp4Tyb mice show a phenotype whereas Dp(16)1Yey/*Dyrk1a*^+/-^ do not, despite having an extra copy of all the genes that are also duplicated in Dp4Tyb. Thus, there must be genes outside the Dp4Tyb region that suppress the effects of increased dosage of gene(s) in Dp4Tyb, and suggests that DS phenotypes may result from the interplay of dosage-sensitive genes that both enhance and suppress phenotypes. DYRK1A is a protein kinase whose overexpression has been proposed to lead to defects in brain development, synaptic plasticity and in learning and memory [28], however, the mechanism by which this happens is not understood.

Recently published studies proposed that increased dosage of regions between *Hspa13* and *App* on Mmu16 and *Abcg1* and *U2af1* on Mmu17 contribute to locomotor defects [13, 40]. These regions are duplicated in Dp9Tyb and Dp(17)1Yey mice respectively and we showed that in three copies they are not sufficient to cause locomotor defects, though we cannot rule out that they could contribute to the phenotype when combined with duplication of the region in Ts1Rhr. Indeed the stronger phenotype in Dp(16)1Yey mice compared to Ts1Rhr may be due to an increased dosage of genes in the Dp9Tyb region.

Previous studies had shown that both Ts65Dn and Tc1 mice have defects in cerebellar anatomy, but despite a more extensive analysis than was carried out in these strains, defects were not observed in Dp(16)1Yey mice. There are a number of differences between these models, including a different complement of duplicated genes, but we note that both Ts65Dn and Tc1 are aneuploid and this may possibly contribute to the phenotype. A recent study reported that Dp(16)1Yey mice have a reduced density of Purkinje cells and granule cells in the cerebellum [41]. It is unclear why our findings are different, but the two studies were carried out on different genetic backgrounds, which may have an effect.

Our results show that both Tc1 and Dp(16)1Yey mice have progressive degenerative loss of motor neurons in the spinal cord. This unexpected and novel phenotype led us to examine spinal cords from humans with DS; importantly, we found that humans also show reduced numbers of motor neurons. Since we only analyzed older adults, we are unable to distinguish if the loss is degenerative or a consequence of developmental abnormalities. To establish this would require analysis of spinal cords from young people with DS; to our knowledge such samples are not currently available. A previous study showed that people with DS have defective peripheral and central nervous system conduction parameters, consistent with axonal degeneration [42]. Interestingly, genetic mapping showed that the Hsa21 orthologous region of Mmu16 was both required and sufficient to cause the motor neuron loss, that the orthologous regions on Mmu10 and Mmu17 did not contribute, and that aneuploidy was not required. However, breaking up the Mmu16 region into three smaller regions caused the phenotype to disappear. Thus, the neuronal loss is caused by at least 2 genes and these must reside in 2 or more of these 3 regions. It is possible that this phenotype is caused by the mass action by an increased dosage of a large number of genes–there are 148 duplicated genes in Dp(16)1Yey–or by a small number of dosage-sensitive genes. Further mapping studies would be needed to distinguish these possibilities.

The extent of motor neuron loss (around 20%) is not large enough to explain the locomotor defects. Furthermore, genetic mapping showed that the locomotor defects and motor neuron loss do not map to the same region, again suggesting that the locomotor defects are not caused by motor neuron loss at the ages we tested. Nonetheless it is possible that the loss of these neurons may contribute to the defects in combination with other pathological mechanisms, and may account for why the locomotor phenotype of Dp(16)1Yey mice is stronger than that of Ts1Rhr mice.

In summary, we have been able to map the genomic location of causative genes underlying DS phenotypes. We have identified that three copies of the *Dyrk1a* gene are required for locomotor dysfunction, a relatively understudied phenotype, and using mouse models of DS we have discovered a decreased number of motor neurons, a novel phenotype which we have shown is also present in humans with DS.

## Materials and methods

### Mice

Mice carrying the Dp(16Lipi-Zbtb21)1TybEmcf (Dp1Tyb), Dp(16Mis18a-Runx1)2TybEmcf (Dp2Tyb), Dp(16Mir802-Zbtb21)3TybEmcf (Dp3Tyb), Dp(16Mir802-Dscr3)4TybEmcf (Dp4Tyb), Dp(16Dyrk1a-B3galt5)5TybEmcf (Dp5Tyb), Dp(16Igsf5-Zbtb21)6TybEmcf (Dp6Tyb), Dp(16Lipi-Hunk)9TybEmcf (Dp9Tyb), Dp(16Lipi-Zbtb21)1Yey (Dp(16)1Yey), Dp(17Abcg1-Rrp1b)1Yey (Dp(17)1Yey), Dp(10Prmt2-Pdxk)1Yey (Dp(10)1Yey), Dp(16Cbr1-Fam3b)1Rhr (Ts1Rhr), Tc(HSA21)1TybEmcf (Tc1) and *Dyrk1a*^tm1Mla^ (*Dyrk1a*^+/-^) alleles have been described [9–11, 16, 27, 43]. Triple trisomic mice were generated by intercrossing Dp(16)1Yey, Dp(10)1Yey and Dp(17)1Yey mice to generate Dp(16)1Yey/Dp(10)1Yey/ Dp(17)1Yey mice with all three duplications. All strains were maintained by backcrossing to C57BL/6JNimr, except for mice bearing Tc1, Ts1Rhr and *Dyrk1a*^+/-^ alleles, which were maintained by crossing to (C57BL/6J x 129S8)F1. All mice on the C57BL/6JNimr background that were used for experiments had been backcrossed for at least 5 generations. The intercross of Dp(16)1Yey and *Dyrk1a*^+/-^ mice was backcrossed to C57BL/6JNimr for two generations. All mice were bred and maintained at the MRC National Institute for Medical Research (now The Francis Crick Institute), except for Dp1Tyb mice used in the Locotronic assay which were bred at the MRC Harwell Institute, and 10-week old Dp(16)1Yey mice whose cerebella were used for Q-RTPCR studies which were bred at Institut de Génétique et de Biologie Moléculaire et Cellulaire, Illkirch, France and provided by Veronique Brault. All experiments were carried out on males, using age-matched littermate controls, except for analysis of Dp(16)1Yey mice at P6 where both genders were used. No randomization was used, but in all cases analyses were carried out by experimenters who were blind to genotype.

### Rotarod

The Rotarod test was used to evaluate locomotor function. 12-week old male mice were habituated to the RotaRod Advanced apparatus (Letica Scientific Instruments) the day before testing commenced by placing them on the rotating rod at a constant speed of 4 rpm. Motor performance was tested by placing the mouse onto the accelerating rod (4 to 40 rpm over 5 min) and then recording the speed of the rod at which the mouse fell off. The trial was repeated three times per day with an hour inter-trial interval. The average RPM of the three trials for each day for each animal was then calculated. Trials were then repeated on a second and third day to evaluate learning.

### Locotronic test

The Locotronic test (Intellibio, France) is a test of locomotor function. Mice traverse a horizontal ladder with evenly spaced rungs, along a narrow corridor to reach the exit. The number of rungs that the mouse stepped on and/or missed was recorded automatically, thereby determining how many rungs were missed. Each animal (n = 13 WT, 12 Dp1Tyb) was tested 2–3 times. Trials where mice took more than 60 s to traverse the ladder were excluded from the analysis; a total of 10 out of 69 trials were excluded, 5 from WT mice and 5 from Dp1Tyb mice, indicating no difference in motivation between genotypes.

### Quantitative real-time PCR

Cerebella from 10-week or 6-day old mice were dissected and snap frozen. Each sample was homogenized in Buffer RLT (Qiagen) with 143mM 2-mercaptoethanol using a Pellet Pestle cordless motor (Kimble). Tissue lysates were loaded onto the QIAshredder (Qiagen) according to manufacturer’s instructions. Total RNA was isolated using RNeasy Mini Kit (Qiagen) and quantified using NanoDrop1000 (Thermo Scientific). RNA was reverse transcribed into cDNA using a MEGAscript T7 Kit (Invitrogen), and analysed by quantitative real-time PCR on a Quant Studio3 Real-Time PCR Machine (Thermo Fisher Scientific) using TaqMan gene expression assays (Thermo Fisher Scientific). Expression of test genes (*Dyrk1a*, *Gad1* and *Gad2*) was normalised to the expression *Gapdh* and then to expression in the wild-type control samples.

### Cerebellar anatomy

P6 and 6-month old mice were sacrificed and brains were extracted and immersion fixed in 10% Formalin (VWR). After fixation, brains were embedded in paraffin and 5 μm sections were taken at the midline. Sections were stained with hematoxylin and eosin and images were acquired using an Olympus VS120 slide scanner. Measurements were performed in FIJI software (ImageJ) and, except where indicated, all enumeration was performed manually using the cell counter tool. Purkinje cell counts were performed along the whole length of the indicated lobule and density derived by dividing cell counts by the length of the Purkinje cell layer. Cerebellar granule cells were counted in identical locations at the tip of each lobule and density calculated by dividing cell counts by the area. Width measurements of the granule cell layer and molecular layer were all performed at identical locations within each lobule. Interneuron numbers were analyzed by drawing regions of interest around the molecular layer of lobule IX and dividing this region into three further regions of interest, then using the automated cell counter with the spot detector plugin in Icy (http://icy.bioimageanalysis.org/). Similarly the cerebellar granule cell numbers at P6 were counted using the automated cell counter in Icy. Tissue samples in this and all other histological analyses were excluded if quality or integrity of the sample was diminished or damaged.

### Sensory tests

#### Cold plate test

Animals were habituated for 1 week prior to the test procedure by restraining the mice and holding a hind paw onto a solid surface as previously described [44]. Following habituation, the solid surface was replaced with a cold plate (10°C). The withdrawal latency for each paw was measured with a cut-off of 1 min and was performed once a day for 3 consecutive days.

#### Hargreaves test of thermal nociception

Heat-pain threshold of the hind paw was ascertained with the Hargreaves method using the Plantar Test (7370; active intensity 20%; Ugo Basile, Italy) [45]. Unrestrained animals were acclimatised in acrylic cubicles (8 × 5 × 10 cm) atop a uniform glass surface for 60 min prior to testing. An infrared light source was directed onto the plantar surface of the hind paw and the latency to paw withdrawal was measured. Four to six responses were recorded for each hind paw on each testing occasion with at least 2 min between stimuli. This was repeated on five consecutive days. To avoid tissue injury, the maximum stimulus latency was 20 s.

#### Von Frey test of mechanosensation

Static mechanical withdrawal thresholds were assessed by applying von Frey hairs (Somedic, Sweden) to the plantar surface of the hind paw. Unrestrained mice were acclimatized in acrylic cubicles (8 × 5 × 10 cm) atop a wire mesh grid for 60 min prior to testing. Calibrated von Frey hairs were applied to the plantar surface of the hind paw until the fiber bent. The 50% withdrawal threshold was determined using the up-down method described previously [46]. The test was performed on both paws once a day for 5 consecutive days.

#### Formalin test

Mice were acclimatized to a Plexiglas container for 30 min and then received an injection of 50 μl of 5% formalin subcutaneously into the plantar surface of the right hind paw. Nocifensive behaviors including paw withdrawal, shaking, biting and licking were manually quantified in 5 min time blocks over the following 1 h. The formalin test was performed once and the data represents mean numbers of total nocifensive behaviors.

#### Beam walk test

This was performed as previously described [47]. Mice were trained for seven days to walk up an inclined tapered beam, and then tested. Scores were assigned for correct steps, with increasing scores on the narrower portions of the beam.

### Immunofluorescence of DRG

Mice were sacrificed and the L3-L5 DRG were dissected and fixed for 90 min in 4% paraformaldehyde in 0.1 M phosphate buffer and mounted in OCT embedding compound. DRG blocks were sectioned on a cryostat at 10 μm thickness. Sections were washed in Phosphate Buffered Saline (PBS) and blocked using 10% normal goat serum (Vector Laboratories) for 30 min. Primary antibodies were incubated overnight and secondary antibodies were incubated for 2 h at room temperature in the dark. After each incubation step, the sections were washed three times with PBS. All reagents were diluted in PBS containing 0.2% Triton X-100 and 0.1% sodium azide. Slides were sealed with coverslips mounted with Vectashield medium. Immunofluorescence was visualized using a Zeiss Imager.ZI fluorescence microscope and pictures acquired with Axiovision software. Primary antibodies or lectins used: polyclonal rabbit anti-mouse CGRP (Biomolecular, CA1137, 1:500), biotinylated isolectin B4 (Sigma, L2140, 1:100), mouse anti-mouse NF200 (Millipore, MAB5266, 1:200), polyclonal rabbit anti-human PGP9.5 (Ultraclone, RA95101, 1:800). Secondary antibodies used: anti-rabbit-Cy3 (Stratech Scientific Ltd. 711-166-152, 1:500), extra-avidin FITC (Invitrogen, 1:400), anti-mouse FITC (Jackson Immunoresearch, 1:500). All antibodies listed here have been validated by their suppliers and references can be found on their website or on the online validation databases Antibodypedia and 1DegreeBio. Quantification was performed by manually counting the total number of DRG cell profiles identified by positive staining for PGP9.5 as well as the numbers of profiles positive for NF200, CGRP or IB4 in a representative region of interest in a DRG, from which a percentage was calculated. For each DRG at least three sections were quantified.

### Physiological assessment of hindlimb muscle function and motor unit number

For the assessment of muscle function *in vivo*, mice were examined as previously described [48].

### Muscle histology

Following the physiological assessment of muscle function, the TA, EDL and soleus muscles were dissected and snap frozen in isopentane cooled in liquid nitrogen and stored at -80°C until processing. Frozen muscle samples were cut on a cryostat at 12 μm. Serial cross sections were collected on glass slides and stained for succinate dehydrogenase (SDH) activity to determine the oxidative capacity of the muscle fibres, as previously described[48].

### Motor neuron counts in mice

In all mouse strains except Tc1 mice at 6 and 19 months, motor neurons were counted using a rapid dissection method. Animals were sacrificed and the L4 and L5 spinal segment was identified externally by distance from the most caudal rib and the iliac crest bone. The whole spinal column was extracted and placed in 10% Formalin before being decalcified by immersion in 5% formic acid (Immunocal, Decal Chemical Corporation, NY, USA) overnight, processed for paraffin embedding and sectioned at 5 μm thickness. Every eighth section was collected, thus giving a 35 μm interval between representative sections and 40 sections in total were collected per animal. Sections were stained with Cresyl Violet for Nissl bodies and images acquired with an Olympus VS120 Slide Scanner. All sections were assessed for correct anatomical location before counting. Motor neurons in the sciatic motor pool were located and counted based on the following criteria: a visible nucleolus, a soma rich in Nissl bodies, a diameter >15 μm and multipolar morphology with visible dendritic branching. Spinal cords of Tc1 mice at 6 and 19 months were removed, fixed in 4% paraformaldehyde and cryopreserved in 30% sucrose overnight. The lumbar L2-L6 region was sectioned on a cryostat and 20 μm transverse sections were collected serially onto glass slides and stained for Nissl bodies with gallocyanin. The number of Nissl-stained large motor neurons in every third L3-L6 lumbar section was established, using the same criteria as described above, with counted sections being at least 60 μm apart. When counting motor neurons at different levels of the spinal cord, the number of sections included in the counts was standardized. Thus, a total of 5 sections from L3, 10 sections from L4, 20 sections from L5 and 5 sections from L6 region were analyzed. 40 sections were counted for each animal.

### Assessment of numbers of spinal motor neurons in human autopsy samples

Cervical spinal cord autopsy samples from people with Down syndrome (n = 3, 58–70 years old), amyotrophic lateral sclerosis (n = 3, 33–70 years old) and controls with no neurological disease (n = 4, 59–71 years old) were obtained from the Netherlands Brain Bank, Netherlands Institute for Neuroscience, Amsterdam, Netherlands (8 samples), and the Thomas Willis Oxford Brain Collection, John Radcliffe Hospital, Oxford UK (3 samples). From the samples, 3 were paraffin-embedded tissues (2 controls and one DS), which were serially cut at 6–14 μm, whereas 8 specimens were frozen tissues which were cut at 20 μm serially. Sections were stained with Luxol Fast Blue, which stains the myelin sheaths blue and combined with Cresyl Violet staining that stains Nissl granules (RNA in rough endoplasmic reticulum) pink. For each sample a total of 20 sections each 60 μm apart, were assessed. The total number of large motor neurons with intense Nissl staining was counted in each section. Results are shown as number of motor neurons per hemisection for each disease group.

### Ethics statement

The work on human tissues was approved by a UK Research Ethics Committee, specifically the NRES Committee London—Queen Square (REC approval number 09/H0716/57). This ethics approval covers the procurement and use of linked-anonymised and anonymised human tissue and samples and data from banks, established collections and collaborators in the UK and internationally. All patient consent, consent from a legal representative, an opinion from a consultee or consent from an individual in a qualifying relationship to the deceased was obtained directly by the providing bank in line with their national legislation and institution policy. In order to maintain donor or consentee privacy, details of personal identifiable information about the patient/donor is held confidentially by the providing bank(s) and was not shared with the research team.

Mouse experiments were approved by the Animal Welfare and Ethical Review Panel (AWERP) of the Francis Crick Institute and were carried out under the authority of a Project Licence (PPL70/8843) granted by the UK Home Office under the regulations of the Animals (Scientific Procedures) Act 1986.

## Supporting information

S1 Table Motor neuron counts in mouse models of DS. Motor neuron counts per 40 sections in mice of indicated genotypes bred from strains with the indicated mutation segregating. All mice were analyzed at 6 months unless otherwise indicated. Triple strain: an intercross of Dp(16)1Yey, Dp(10)1Yey and Dp(17)1Yey, generating 8 different genotypes, of which motor neurons were counted in WT, Dp(16)1Yey and triple mutants (Dp(16)1Yey/Dp(10)1Yey/Dp(17)1Yey). WT, wild-type.
(DOCX)

S2 Table Motor neuron counts in humans with DS. Motor neuron counts per hemisection of cervical spinal cord of people with DS or ALS, or controls who had neither condition.
(DOCX)
