# Supplementary material for: Analysis of motor dysfunction in Down Syndrome reveals motor neuron degeneration
Source: PLoS Genet. 2018 May 10;14(5):e1007383. doi: 10.1371/journal.pgen.1007383 (PMC5963810; doi:10.1371/journal.pgen.1007383)
Supplement: S1 Table — Motor neuron counts per 40 sections in mice of indicated genotypes bred from strains with the indicated mutation segregating. All mice were analyzed at 6 months unless otherwise indicated. Triple strain: an intercross of Dp(16)1Yey, Dp(10)1Yey and Dp(17)1Yey, generating 8 different genotypes, of which motor neurons were counted in WT, Dp(16)1Yey and triple mutants (Dp(16)1Yey/Dp(10)1Yey/Dp(17)1Yey). WT, wild-type. (DOCX) [file pgen.1007383.s001.docx]

**Supplementary Table 1**

| **Strain** | **Genotype** | **Motor neuron counts/40 sections**  **Mean ± SEM** |
| --- | --- | --- |
| Tc1  (P22) | WT | 554.3 ± 24.4 |
|  | Tc1 | 617.5 ± 29.0 |
| Tc1 | WT | 409.4 ± 6.7 |
|  | Tc1 | 337.4 ± 11.2 |
| Tc1  (19 months) | WT | 336.4 ± 7.2 |
|  | Tc1 | 257.8 ± 13.5 |
| Dp(10)1Yey | WT | 411.6 ± 9.5 |
|  | Dp(10)1Yey | 408.9 ± 8.8 |
| Dp(17)1Yey | WT | 396.7 ± 12.5 |
|  | Dp(17)1Yey | 380.8 ±13.4 |
| Triple | WT | 379.4 ± 16.1 |
|  | Dp(16)1Yey | 303.4 ± 22.2 |
|  | Triple | 332.9 ± 16.8 |
| Dp(16)1Yey  (P6) | WT | 383.8 ± 13.2 |
|  | Dp(16)1Yey | 390.0 ± 10.1 |
| Dp2Tyb | WT | 385.7 ± 10.6 |
|  | Dp2Tyb | 380.3 ± 11.8 |
| Dp3Tyb | WT | 363.5 ± 20.9 |
|  | Dp3Tyb | 362.9 ± 21.6 |
| Dp9Tyb | WT | 382.2 ± 13.7 |
|  | Dp9Tyb | 378.9 ± 21.4 |
