# Supplementary material for: Analysis of motor dysfunction in Down Syndrome reveals motor neuron degeneration
Source: PLoS Genet. 2018 May 10;14(5):e1007383. doi: 10.1371/journal.pgen.1007383 (PMC5963810; doi:10.1371/journal.pgen.1007383)
Supplement: S2 Table — Motor neuron counts per hemisection of cervical spinal cord of people with DS or ALS, or controls who had neither condition. (DOCX) [file pgen.1007383.s002.docx]

**Supplementary Table 2**

| **Condition** | **Motor neuron counts/hemisection**  **Mean ± SEM** |
| --- | --- |
| Control | 14.2±0.5 |
| DS | 10.6±0.4 |
| ALS | 8.0±0.5 |
